# Supplementary material for: From sequence to enzyme mechanism using multi-label machine learning
Source: BMC Bioinformatics. 2014 May 19;15:150. doi: 10.1186/1471-2105-15-150 (PMC4229970; doi:10.1186/1471-2105-15-150)
Supplement: Additional file 2 — Java code of ml2db. Additional file ml2db_code.tar.gz contains the Java source code to run the multi-label machine learning experiments and save the results to database. The code’s Javadoc is included. [file 1471-2105-15-150-S2.zip › additional file 2/ml2db/ml2db/doc/mulan/classifier/transformation/PrunedSets.html]

PrunedSets


JavaScript is disabled on your browser.


- Overview
- Package
- Class
- Use
- Tree
- Deprecated
- Index
- Help

- Prev Class
- Next Class

- Frames
- No Frames

- All Classes

- Summary:
- Nested |
- Field |
- Constr |
- Method

- Detail:
- Field |
- Constr |
- Method


mulan.classifier.transformation

## Class PrunedSets

- java.lang.Object
- - mulan.classifier.MultiLabelLearnerBase
  - - mulan.classifier.transformation.TransformationBasedMultiLabelLearner
    - - mulan.classifier.transformation.LabelPowerset
      - - mulan.classifier.transformation.LabelsetPruning
        - - mulan.classifier.transformation.PrunedSets

- All Implemented Interfaces:
  :   java.io.Serializable, MultiLabelLearner, weka.core.TechnicalInformationHandler

  ---

    

  ```
  public class PrunedSets
  extends LabelsetPruning
  ```

  Class implementing the Pruned Sets algorithm (PS). For more information, see

  Version:
  :   2012.02.27

  Author:
  :   Grigorios Tsoumakas

  See Also:
  :   Serialized Form

- - ### Nested Class Summary

    Nested Classes

    | Modifier and Type | Class and Description |
    | --- | --- |
    | `static class` | `PrunedSets.Strategy` strategies for processing infrequent labelsets |
  - ### Constructor Summary

    Constructors

    Constructor and Description

    `PrunedSets()`

    Default constructor

    `PrunedSets(weka.classifiers.Classifier classifier,
    int aP,
    PrunedSets.Strategy aStrategy,
    int aB)`

    Constructor that initializes learner with base algorithm, parameter p
    and strategy for processing infrequent labelsets
  - ### Method Summary

    Methods

    | Modifier and Type | Method and Description |
    | --- | --- |
    | `weka.core.TechnicalInformation` | `getTechnicalInformation()` Returns an instance of a TechnicalInformation object, containing detailed information about the technical background of this class, e.g., paper reference or book this class is based on. |
    | `java.lang.String` | `globalInfo()` Returns a string describing classifier |

    - ### Methods inherited from class mulan.classifier.transformation.LabelPowerset

      `setConfidenceCalculationMethod, setMakePredictionsBasedOnConfidences, setSeed, setThreshold`
    - ### Methods inherited from class mulan.classifier.transformation.TransformationBasedMultiLabelLearner

      `getBaseClassifier`
    - ### Methods inherited from class mulan.classifier.MultiLabelLearnerBase

      `build, getDebug, isUpdatable, makeCopy, makePrediction, setDebug`
    - ### Methods inherited from class java.lang.Object

      `equals, getClass, hashCode, notify, notifyAll, toString, wait, wait, wait`

- - ### Constructor Detail


    - #### PrunedSets

      ```
      public PrunedSets()
      ```

      Default constructor


    - #### PrunedSets

      ```
      public PrunedSets(weka.classifiers.Classifier classifier,
                int aP,
                PrunedSets.Strategy aStrategy,
                int aB)
      ```

      Constructor that initializes learner with base algorithm, parameter p
      and strategy for processing infrequent labelsets

      Parameters:
      :   `classifier` - base single-label classification algorithm
      :   `aP` - number of instances required for a labelset to be included.
      :   `aStrategy` - strategy for processing infrequent labelsets
      :   `aB` - parameter of the strategy for processing infrequent labelsets
  - ### Method Detail


    - #### getTechnicalInformation

      ```
      public weka.core.TechnicalInformation getTechnicalInformation()
      ```

      **Description copied from class: `TransformationBasedMultiLabelLearner`**

      Returns an instance of a TechnicalInformation object, containing detailed
      information about the technical background of this class, e.g., paper
      reference or book this class is based on.

      **Specified by:**
      :   `getTechnicalInformation` in interface `weka.core.TechnicalInformationHandler`

      **Overrides:**
      :   `getTechnicalInformation` in class `TransformationBasedMultiLabelLearner`

      Returns:
      :   the technical information about this class


    - #### globalInfo

      ```
      public java.lang.String globalInfo()
      ```

      Returns a string describing classifier

      **Overrides:**
      :   `globalInfo` in class `TransformationBasedMultiLabelLearner`

      Returns:
      :   a description suitable for displaying


- Overview
- Package
- Class
- Use
- Tree
- Deprecated
- Index
- Help

- Prev Class
- Next Class

- Frames
- No Frames

- All Classes

- Summary:
- Nested |
- Field |
- Constr |
- Method

- Detail:
- Field |
- Constr |
- Method
